# Assessment of the Plasmodium falciparum Preerythrocytic Antigen UIS3 as a Potential Candidate for a Malaria Vaccine

**DOI:** 10.1128/IAI.00641-16

**Published:** 2017-02-23

**Authors:** Rhea J. Longley, Benedict R. Halbroth, Ahmed M. Salman, Katie J. Ewer, Susanne H. Hodgson, Chris J. Janse, Shahid M. Khan, Adrian V. S. Hill, Alexandra J. Spencer

**Affiliations:** a The Jenner Institute, University of Oxford, Oxford, United Kingdom; b Department of Parasitology, Leiden University Medical Center, Leiden, The Netherlands; University of South Florida

**Keywords:** Plasmodium, UIS3, liver stage, malaria, sterile protection, synergy, vaccines

## Abstract

Efforts are under way to improve the efficacy of subunit malaria vaccines through assessments of new adjuvants, vaccination platforms, and antigens. In this study, we further assessed the Plasmodium falciparum antigen upregulated in infective sporozoites 3 (PfUIS3) as a vaccine candidate. PfUIS3 was expressed in the viral vectors chimpanzee adenovirus 63 (ChAd63) and modified vaccinia virus Ankara (MVA) and used to immunize mice in a prime-boost regimen. We previously demonstrated that this regimen could provide partial protection against challenge with chimeric P. berghei parasites expressing PfUIS3. We now show that ChAd63-MVA PfUIS3 can also provide partial cross-species protection against challenge with wild-type P. berghei parasites. We also show that PfUIS3-specific cellular memory responses could be recalled in human volunteers exposed to P. falciparum parasites in a controlled human malaria infection study. When ChAd63-MVA PfUIS3 was coadministered with the vaccine candidate P. falciparum thrombospondin-related adhesion protein (PfTRAP) expressed in the ChAd63-MVA system, there was no significant change in immunogenicity to either vaccine. However, when mice were challenged with double chimeric P. berghei-P. falciparum parasites expressing both PfUIS3 and PfTRAP, vaccine efficacy was improved to 100% sterile protection. This synergistic effect was evident only when the two vaccines were mixed and administered at the same site. We have therefore demonstrated that vaccination with PfUIS3 can induce a consistent delay in patent parasitemia across mouse strains and against chimeric parasites expressing PfUIS3 as well as wild-type P. berghei; when this vaccine is combined with another partially protective regimen (ChAd63-MVA PfTRAP), complete protection is induced.

## INTRODUCTION

Plasmodium falciparum remains the leading causative agent of mortality due to human malaria, and eradication of this disease is a leading public health goal in many developing countries. Vaccination is considered to be a cost-effective preventative health tool and is considered vitally important for elimination of this disease (1). The leading malaria vaccine currently undergoing assessment in areas where malaria is endemic is RTS,S/AS01 (2), a subunit vaccine encoding the preerythrocytic antigen circumsporozoite protein (CSP). While this vaccine has proven to be partially effective (3), efforts continue to increase durable efficacy through assessments of new adjuvants, new delivery platforms, and/or new candidate antigens.

Our past research demonstrated the capacity of viral vectors, as delivery platforms, to induce high-magnitude antigen-specific cellular immune responses in both animal models (4) and humans (5). Cellular immunity is essential for targeting the liver stage of the parasite's life cycle (6). A prime-boost regimen using the viral vectors chimpanzee adenovirus 63 (ChAd63) and modified vaccinia virus Ankara (MVA) has so far proved to be the most adept at inducing high-magnitude cellular immunity (7). Use of this regimen with vectors encoding the thrombospondin-related adhesion protein (TRAP) along with a multiepitope (ME) string resulted in moderate efficacy against P. falciparum sporozoites in malaria-naive adults (5) and in a field trial in a region where malaria is endemic (8). Building upon this work, we recently screened eight new virally vectored vaccines (containing P. falciparum preerythrocytic antigens as inserts) and compared their efficacies in mice against that induced by CSP or TRAP (9). We identified two antigens, P. falciparum liver-stage antigen 1 (PfLSA1) and liver-stage-associated protein 2 (PfLSAP2), that provided superior protection against challenge with chimeric P. berghei-P. falciparum parasites expressing the cognate P. falciparum antigen (9).

In addition to PfLSA1 and PfLSAP2, we observed that immunization of mice with viral vectors expressing the antigen upregulated in infective sporozoites 3 (UIS3) provided protection in BALB/c mice equal to that obtained by immunization with similar vectors expressing PfCSP. PfUIS3 did not induce protection in outbred CD-1 mice, but there was a median 1.1-day delay in the time to patent parasitemia (9). PfUIS3 is a 229-amino-acid protein that is a member of the early transcribed membrane protein (ETRAMP) family (10). PfUIS3 is hence also known as ETRAMP13 and is relatively conserved, with orthologs in P. yoelii, P. vivax, P. cynomolgi, P. reichenowi, P. berghei, and P. chabaudi, according to the PlasmoDB database (plasmodb.org). UIS3 became a protein of interest when it was shown to be upregulated in salivary gland sporozoites compared to oocyst sporozoites (PbUIS3) (11), and it has since been demonstrated that expression of this protein is restricted to sporozoites and liver-stage parasites for P. yoelii (12, 13), with no expression during the blood stage. Kaiser and colleagues (12) were also able to compare their PyUIS3 expression results with those of a P. falciparum microarray expression study (14), and they identified that PfUIS3 was also highly upregulated in sporozoites compared to asexual blood-stage parasites. This confirms an earlier report that PfUIS3 is not expressed during blood stages (10). UIS3 was subsequently shown to be essential for early liver-stage development in P. berghei (15) and P. yoelii (16). Parasites without UIS3 can still invade liver cells but fail to develop into mature liver-stage schizonts and fail to reach the blood stage. PfUIS3 is predicted to have an N-terminal signal peptide and two transmembrane domains (the first overlapping the predicted signal peptide) (see Fig. S1 in the supplemental material), suggesting that it may be localized to the membrane. Indeed, evidence of localization to the parasitophorous vacuole membrane (PVM) has been demonstrated for PyUIS3 (17). While the function of UIS3 is still unclear, the protein likely has a role in the importation of fatty acids into the PVM (17, 18). Utilizing PyUIS3 in a mouse liver model, it was shown that this protein directly interacts with the liver-fatty acid binding protein (L-FABP). L-FABPs have a known role in facilitating the utilization of fatty acids in liver cells (19), and it was demonstrated that levels of L-FABP in host hepatocytes directly correlated with liver-stage parasite growth (17). There is also evidence that PfUIS3 interacts directly with human L-FABP (18), supporting this proposed function. In addition to our recent work, PyUIS3 has been shown to provide protection against malaria when administered in combination with PyFalstatin. Using a vaccination regimen of DNA followed by a replication-competent vaccinia virus vector, these antigens together resulted in 43% sterile protection in outbred mice (13).

In the current study, we aimed to further investigate the potential of PfUIS3 as a vaccine candidate. We demonstrate here that CD8^+^ T cells are critical for protection induced by ChAd63-MVA PfUIS3, providing further evidence that this vaccine targets PfUIS3 expressed in liver-stage parasites. We also show that PfUIS3 is capable of inducing protection not only against chimeric P. berghei-P. falciparum parasites expressing PfUIS3 but also against wild-type (WT) P. berghei parasites. In addition, we compared cellular and humoral immune responses of mice immunized with PfUIS3 either alone or in combination with PfTRAP, and we found no significant differences. Furthermore, when both PfUIS3 and PfTRAP were coadministered as a mixture, we observed 100% sterile protection against challenge with double chimeric P. berghei-P. falciparum parasites expressing both PfUIS3 and PfTRAP. We also showed that PfUIS3 memory responses could be recalled in human volunteers recently exposed to P. falciparum parasites. Together, our results suggest that PfUIS3 may be considered for inclusion in a multiantigen subunit vaccine.

## RESULTS

### PfUIS3 can provide protection against challenge with chimeric P. berghei-P. falciparum parasites in multiple strains of mice.

**FIG 1:**
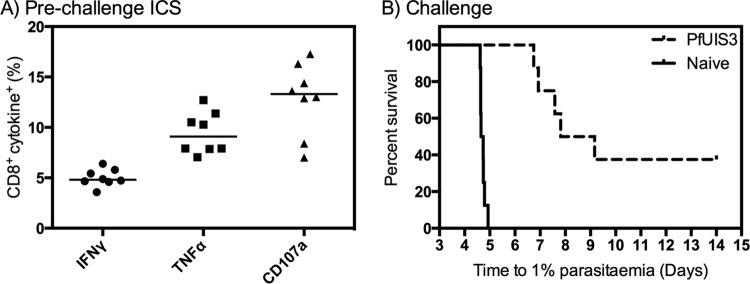
PfUIS3 protects C57BL/6 mice against chimeric P. berghei-P. falciparum challenge. C57BL/6 mice (*n* = 8) were vaccinated i.m. with 1 × 10^8^ inclusion-forming units (IFU) of ChAd63-PfUIS3 followed 8 weeks later by 6 × 10^6^ PFU of MVA-PfUIS3. (A) Cellular immunogenicity was assessed in the blood at 7 days postboost by ICS after stimulation for 6 h with an overlapping peptide pool. Both medians and individual data points are shown. (B) At 10 days postboost, mice were challenged i.v. with 1,000 chimeric P. berghei-P. falciparum sporozoites expressing PfUIS3, as were eight naive control mice. The log-rank (Mantel-Cox) test was used to assess the difference between the survival curves (*P* < 0.0001).

We previously demonstrated that PfUIS3, administered in a ChAd63-MVA prime-boost regimen with an 8-week interval, could induce protective immune responses in BALB/c mice as determined by a significant delay in the time to 1% parasitemia after challenge with chimeric P. berghei-P. falciparum parasites expressing PfUIS3 (9). We also identified conserved immunogenic epitopes for PfUIS3 in both BALB/c and C57BL/6 mice (20). Therefore, we initially wished to determine whether PfUIS3 could induce protective immunity in C57BL/6 mice, an additional strain of inbred mice. C57BL/6 mice were vaccinated with ChAd63-MVA PfUIS3, using an 8-week interval, followed 10 days later by challenge with 1,000 chimeric P. berghei-P. falciparum sporozoites injected intravenously (i.v.). Vaccination of C57BL/6 mice induced a strong proinflammatory response in the blood, with a median of 4.8% CD8^+^ IFN-γ^+^ cells (Fig. 1A), and provided a significant delay in the time to 1% parasitemia while also sterilely protecting 3/8 mice (*P* < 0.0001 by the log-rank [Mantel-Cox] test) (Fig. 1B). This experiment was repeated, and again, PfUIS3 vaccination provided a significant delay in the time to 1% parasitemia, and it sterilely protected 2/8 mice (*P* < 0.0001) (data not shown).

### PfUIS3 can provide protection against WT P. berghei challenge.

**FIG 2:**
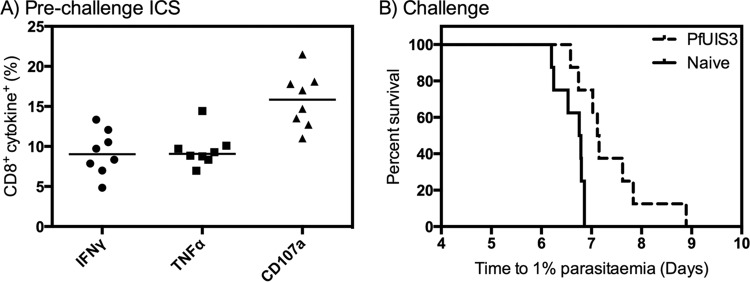
PfUIS3 protects BALB/c mice against WT P. berghei challenge. BALB/c mice (*n* = 8) were vaccinated i.m. with 1 × 10^8^ IFU of ChAd63-PfUIS3 followed 8 weeks later by 1 × 10^7^ PFU of MVA-PfUIS3. (A) Cellular immunogenicity was assessed in the blood at 6 days postboost by ICS after stimulation for 6 h with an overlapping peptide pool. Both medians and individual data points are shown. (B) At 8 days postboost, mice were challenged i.v. with 1,000 WT P. berghei sporozoites, as were eight naive control mice. The log-rank (Mantel-Cox) test was used to assess the difference between the survival curves (*P* = 0.0048).

The UIS3 gene is present in both the P. falciparum and P. berghei genomes. Using the European Bioinformatics Institute EMBOSS Needle pairwise protein sequence alignment tool (http://www.ebi.ac.uk/Tools/psa/emboss_needle/), we found 54% sequence similarity and 33% identity between the P. falciparum and P. berghei UIS3 sequences. Given that previous research demonstrated the feasibility of cross-species protection using subunit vaccines (21, 22), we next assessed whether PfUIS3 could provide protection of BALB/c mice against challenge with WT P. berghei sporozoites. A moderate immune response was detected in BALB/c mice, as expected (9), with a median of 9.0% CD8^+^ IFN-γ^+^ cells in the blood (Fig. 2A). Upon challenge with 1,000 WT P. berghei sporozoites at 8 days post-MVA boost, there was a statistically significant delay in the time to reach 1% parasitemia in vaccinated mice compared to naive controls (median delay, 0.36 day; *P* = 0.0048 by the log-rank [Mantel-Cox] test) (Fig. 2B). No mice were sterilely protected. In comparison, no protection against WT P. berghei challenge was observed in mice that were immunized with either of two other candidate antigens with similar levels of sequence similarity and identity between the P. falciparum and P. berghei orthologs (CelTOS, 65% and 45%, respectively; and Falstatin, 53% and 35%, respectively) (see Fig. S6 in the supplemental material).

### PfUIS3 likely provides protection through antigen-specific CD8^+^ T cells.

**FIG 3:**
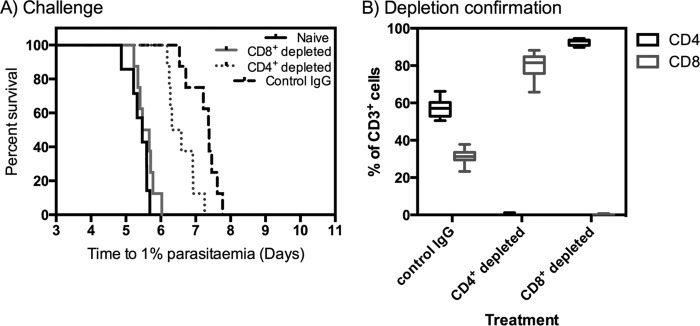
PfUIS3 requires CD8^+^ T cells to provide protection against chimeric P. berghei-P. falciparum challenge in BALB/c mice. BALB/c mice (*n* = 3 groups of 8) were vaccinated i.m. with 1 × 10^8^ IFU of ChAd63-PfUIS3 followed 8 weeks later by 5 × 10^6^ PFU of MVA-PfUIS3. Mice were then injected intraperitoneally (i.p.) with 100 μg of MAb to either CD4^+^ (GK1.5) or CD8^+^ (8.43) or with an IgG MAb control at days 8, 9, and 10 postboost. (A) At day 10, all mice were challenged i.v. with 1,000 chimeric P. berghei-P. falciparum sporozoites expressing PfUIS3, as were seven naive control mice. The log-rank (Mantel-Cox) test was used to assess differences between the survival curves: for CD8^+^ cell-depleted versus naive mice, *P* = 0.13; for CD8^+^ cell-depleted versus control IgG-treated mice, *P* < 0.0001; for CD4^+^ cell-depleted versus naive mice, *P* < 0.0001; and for CD4^+^ cell-depleted versus IgG control-treated mice, *P* = 0.0044. (B) Depletion of CD8^+^ or CD4^+^ T cells was confirmed in the blood at day 4 with respect to challenge. Cells were stained with anti-CD4–FITC clone RM4-5, 1/200 anti-CD8–PerCP-Cy5.5 clone 53-6.7, and 1/50 anti-CD3ε–APC, and the proportion of CD3^+^ cells that were CD4^+^ or CD8^+^ is shown.

Because UIS3 is expressed during both the sporozoite and liver stages (15, 17) and vaccination induces both cellular and humoral immune responses (9), we next wished to determine the mechanism of protection observed in BALB/c mice. We depleted either CD4^+^ or CD8^+^ T cells from PfUIS3-vaccinated BALB/c mice prior to challenge with 1,000 chimeric P. berghei-P. falciparum parasites expressing PfUIS3. Depletion of CD8^+^ T cells completely abolished the protective effect of the vaccine, with no significant difference in the times to reach 1% parasitemia between this group of mice and naive controls and a significant difference compared to mice injected with an IgG control (*P* < 0.0001 by the log-rank [Mantel-Cox] test) (Fig. 3A). The median time to 1% parasitemia was reduced from 7.37 days for the IgG control mice to 5.57 days for CD8^+^ cell-depleted mice. CD4^+^ depletion also significantly reduced the efficacy compared to that with IgG control mice (*P* = 0.0044); however, this regimen still provided some degree of protection compared to naive mice (*P* < 0.0001). We previously identified a conserved epitope in BALB/c, C57BL/6, and HLA-A2-transgenic mice that elicited a CD4^+^ T cell response (20); this may be an important feature of PfUIS3 for the consistency in protection across mouse strains that we observed. We confirmed that either CD4^+^ or CD8^+^ T cells remained depleted 4 days following challenge (Fig. 3B).

**FIG 4:**
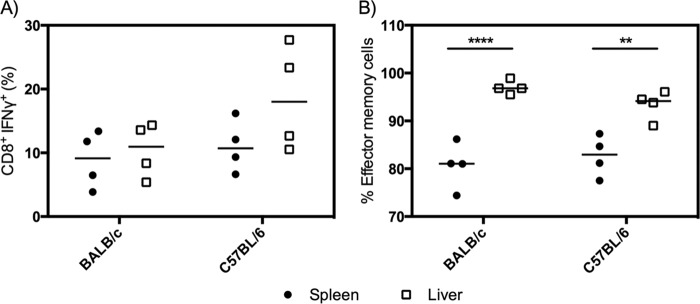
PfUIS3 vaccination induces antigen-specific effector memory cells in the livers of both BALB/c and C57BL/c mice. BALB/c or C57BL/6 mice (*n* = 4 per strain) were vaccinated i.m. with 1 × 10^8^ IFU of ChAd63-PfUIS3 followed 8 weeks later by 1 × 10^6^ PFU of MVA-PfUIS3. Cellular immunogenicity was assessed in both the spleen and liver at 2 weeks postboost. (A) Percentages of CD8^+^ cells secreting IFN-γ in each organ. Closed circles, spleen; open squares, liver. (B) Percentages of antigen-specific cells in each organ that were of the effector memory subtype. Both medians and individual data points are shown. Differences between the spleen and liver for each mouse strain were assessed using two-way ANOVA with Sidak's multiple-comparison test. For panel A, the differences were not significant; for panel B, *P* < 0.0001 for BALB/c mice and *P* = 0.0027 for C57BL/6 mice.

We also observed PfUIS3-specific CD8^+^ T cells in the livers of vaccinated BALB/c and C57BL/6 mice, with a magnitude equal to or greater than that observed in the spleen (Fig. 4A). The majority of PfUIS3-specific cells (defined by gamma interferon [IFN-γ] secretion) in the liver were of the effector memory subtype (CD62L^−^ CD127^+^) (Fig. 4B). For both BALB/c and C57BL/6 mice, a significantly larger proportion of antigen-specific CD8^+^ cells were of the effector memory subset in the liver than in the spleen (*P* < 0.0001 and *P* = 0.0027, respectively, by two-way analysis of variance [ANOVA]).

### Coadministration of ChAd63-MVA PfUIS3 with the vaccine ChAd63-MVA ME-TRAP results in 100% sterile protection in BALB/c mice.

**FIG 5:**
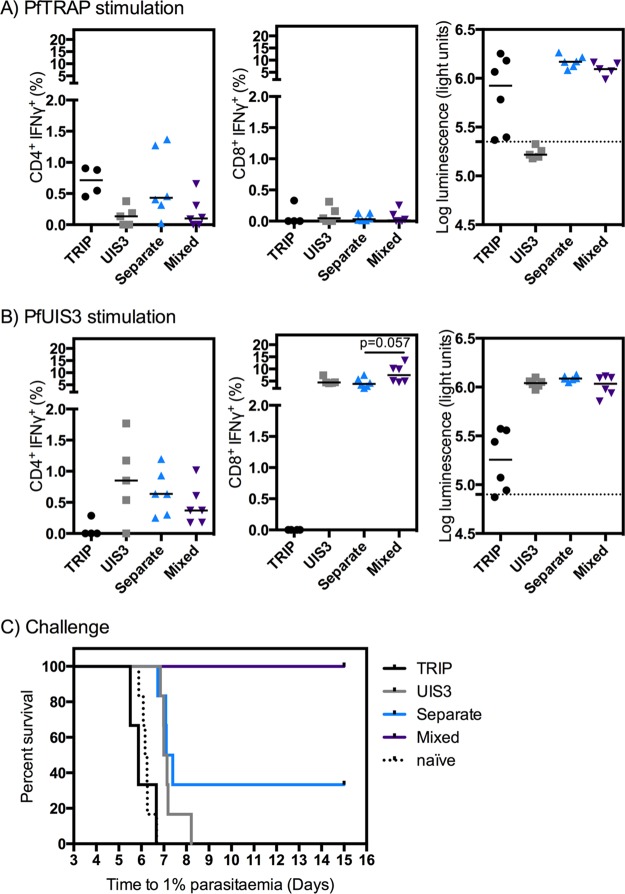
Coadministration of ChAd63-MVA PfUIS3 with the vaccine ChAd63-MVA TRIP is not detrimental to immunogenicity and increases efficacy in BALB/c mice. BALB/c mice (*n* = 3 to 6 per group) were vaccinated i.m. with 1 × 10^8^ IFU of ChAd63 followed 8 weeks later by 1 × 10^7^ PFU of MVA, with the antigens indicated on the *x* axis. When two vaccines were given, mice were vaccinated with a full dose of each vaccine administered in separate legs (separate), or the vaccines were mixed prior to administration in both legs (mixed). Cellular immunogenicity in the blood and IgG responses in plasma were assessed at 1 week postboost. Cells or plasma was incubated with a peptide pool or protein lysate for either PfTRAP (A) or PfUIS3 (B). Both medians and individual data points are shown. The Kruskal-Wallis test with Dunn's multiple-comparison test was used to assess statistical differences between coadministered vaccines and those given alone; no significant differences were found. (C) The same mice were then challenged at 10 days postboost with 1,000 double chimeric P. berghei-P. falciparum sporozoites that expressed both PfTRAP and PfUIS3. The log-rank (Mantel-Cox) test was used to determine the statistical difference between survival curves: for the PfTRIP versus naive groups, the difference was not significant (*P* = 0.5); for the PfUIS3 versus naive groups, *P* = 0.0005; for the PfUIS3 versus separate groups, the difference was not significant (*P* = 0.26); and for the PfUIS3 versus mixed groups, *P* = 0.0005.

ME-TRAP is one of the leading preerythrocytic P. falciparum vaccine candidates (5). We wanted to determine if there was any impact on immunogenicity when PfUIS3 was coadministered with a PfTRAP-based vaccine. As described in Materials and Methods, we chose to use the vaccine PfTRIP, as this uses the TRAP sequence of P. falciparum strain 3D7, the same strain used for the UIS3 sequence. We observed no statistically significant differences in cellular or humoral immune response as measured by blood intracellular cytokine staining (ICS) or luminescence immunoprecipitation system (LIPS) assay between BALB/c mice immunized with the two vaccines administered together and mice immunized with either vaccine alone (Fig. 5). There was a trend toward a lower PfTRAP-specific CD4^+^ IFN-γ^+^ response when the PfTRIP vaccine was coadministered with PfUIS3 (mixed); however, this was not statistically significant. Conversely, coadministration (whether at separate sites or mixed) resulted in slightly higher TRAP-specific IgG antibody responses than those obtained with PfTRIP administered alone (Fig. 5A). There was a higher level of variation in the TRAP-specific IgG response between mice following single TRIP vaccination than following coadministration; this likely was due to experimental variation and may account for the trend toward higher responses in the UIS3/TRAP coadministration groups. The pattern was similar for PfUIS3: slightly lower CD4^+^ IFN-γ^+^ responses were observed following coadministration, but CD8^+^ IFN-γ^+^ and antibody responses were higher than or equal to those with the vaccine administered alone (Fig. 5B).

To assess the protective efficacy of combination vaccination, we generated a double chimeric P. berghei-P. falciparum parasite line expressing both PfUIS3 and PfTRAP (Fig. S2 and S3). The P. falciparum *trap* and *uis3* coding sequences (CDS) were introduced as additional copies in two neutral loci in the P. berghei genome (*Pb230p* and *Pbs1*). Both CDS are under the control of the *Pbuis4* regulatory sequences to drive expression in sporozoites and liver-stage parasites (9). Despite the non-statistically significant immunological differences with separate and mixed PfUIS3 and PfTRIP vaccinations, these immunized mice showed improved protective efficacy against challenge with the double chimeric P. berghei-P. falciparum parasites (Fig. 5C). While PfUIS3 alone provided a delay in the time to 1% parasitemia, as expected, coadministration of PfUIS3 with PfTRIP at the same site resulted in 100% sterile protection (a significant improvement compared to the result with PfUIS3 alone [*P* = 0.0005 by the log-rank {Mantel-Cox} test] or compared to TRIP alone). There was no statistically significant improvement in efficacy when mice were immunized with both PfUIS3 and PfTRIP with delivery of the vaccines at separate sites (*P* = 0.26 compared to PfUIS3 alone) (Fig. 5C).

### PfUIS3-specific memory responses are induced in human volunteers who undergo CHMI with P. falciparum sporozoites.

**FIG 6:**
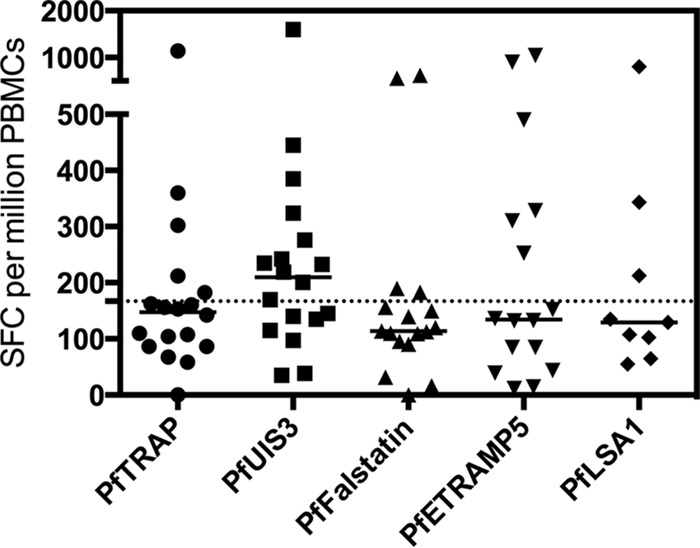
PfUIS3-specific memory responses were induced in human volunteers who had recently undergone CHMI with cryopreserved P. falciparum sporozoites. Frozen PBMCs from 18 volunteers 35 days after CHMI were stimulated for 10 days with a pool of overlapping peptides to one of five preerythrocytic antigens (PfTRAP, PfUIS3, PFI0580c, PFE1590w, and PfLSA1) and recombinant human IL-2. For some volunteers, there were not enough PBMCs available to perform stimulations with all five antigens. The stimulated cells were then used in a standard IFN-γ ELISpot assay, and samples were tested in quadruplicate. Responses to antigens are expressed as numbers of spot-forming cells (SFC) per million original PBMCs. The positivity cutoff (167 SFC) was set using PBMCs from four volunteers from the day before CHMI, as described in Materials and Methods. Medians and individual data points are shown.

We previously reported that modest P. falciparum-specific cellular responses are detectable following controlled human malaria infection (CHMI) with cryopreserved P. falciparum sporozoites (23). We did not identify one antigen that was immunodominant among the 16 tested, but each antigen did induce a modest response in at least one volunteer at one time point. As these *ex vivo* responses were of low magnitude, we aimed to determine the presence of PfUIS3-specific T cell central memory responses by using a cultured IFN-γ-specific enzyme-linked immunosorbent spot (ELISpot) assay (24). Frozen peripheral blood mononuclear cells (PBMCs) collected from 18 volunteers 35 days after CHMI with Sanaria PfSPZ challenge were stimulated for 10 days with recombinant human interleukin-2 (IL-2) and the appropriate peptide pool. Responses to PfUIS3 and four other preerythrocytic antigens (PfTRAP, PfFalstatin, PfLSA1, and PfETRAMP5) were assessed. In cases where sufficient PBMCs were available, responses were measured for all 18 volunteers, regardless of whether they were diagnosed with malaria (four volunteers did not develop blood-stage infection, as demonstrated by persistently negative quantitative PCR [qPCR] results for blood post-CHMI [23]). This was because the four volunteers who were not diagnosed with blood-stage malaria did demonstrate responses to preerythrocytic and blood-stage antigens in the *ex vivo* ELISpot assay (23; R. J. Longley and A. J. Spencer, unpublished data). Among the responses to all the antigens, only the median response to PfUIS3 fell above a positivity threshold set based upon pre-CHMI background levels, although there were no statistically significant differences in the responses to all five antigens (*P* = 0.51 by the Kruskal-Wallis test with Dunn's multiple-comparison test) (Fig. 6). If the four undiagnosed volunteers were excluded, the median response to PfUIS3 still remained higher than those to the other antigens; however, the median response to PfETRAMP5 also exceeded the positivity threshold in this case (data not shown).

## DISCUSSION

Immunization with PfUIS3 administered in the viral vectors ChAd63 and MVA is unable to confer high levels of sterile protection in mice (9). However, we now demonstrate that protective immune responses can be induced in two strains of inbred mice, as shown by a consistent delay of more than 1 day in the time to 1% parasitemia after challenge with chimeric P. berghei-P. falciparum parasites expressing PfUIS3. We observed a greater level of efficacy in C57BL/6 mice than in BALB/c mice, with a 2.9-day median delay in the time to 1% parasitemia versus 1.4 days, even though C57BL/6 mice are conventionally considered more difficult to protect (25). Furthermore, after immunization with PfUIS3 in our previous study, we also observed a 1.1-day median delay in the time to 1% parasitemia in outbred mice that were challenged with the same chimeric P. berghei-P. falciparum parasites (although this was not statistically significant) (9). We show here that vaccination with PfUIS3 can also induce comparable levels of protection against challenge of BALB/c mice with WT P. berghei sporozoites, which express only PbUIS3. This is one of only a few reported cases of cross-species protection induced by malaria vaccines, in particular using subunit vaccination (21, 22, 26–30), and suggests that this vaccination regimen might be capable of providing some degree of strain-transcending immunity (at least against P. berghei). It was beyond the scope of the current study to determine the mechanism of cross-species protection; however, given the lack of long (8-mer) stretches of conserved residues between PfUIS3 and PbUIS3, it is possible that the protection against WT challenge is mediated by antibodies rather than cellular responses. The mechanism will be investigated in future research through the use of passive transfer experiments and mapping of cellular responses against PbUIS3.

Evidence suggests that protective immune responses after immunization with whole, attenuated parasites are dependent on (31, 32), or at least associated with (33, 34), liver-resident effector memory CD8^+^ T cells secreting IFN-γ. As the collection and analysis of liver-resident T cells are not possible in human trials, assessment of correlates of protection has largely been performed on T cells in the periphery. Studies of mice have shown that immunization with viral vectors expressing ME-TRAP induces high percentages of antigen-specific CD8^+^ T cells in the liver prior to sporozoite challenge (35, 36). Upon challenge, this enables a fast and efficient immune response to be mounted, which may be critical given the relatively short time that the parasite spends in the liver. Following PfUIS3 immunization, we observed a high percentage (10 to 20%) of PfUIS3-specific CD8^+^ T cells secreting IFN-γ in the liver for both BALB/c and C57BL/6 mice. By analysis of protective efficacy in immunized BALB/c mice that were depleted of T cells prior to challenge, we were also able to demonstrate that CD8^+^ T cells are required for protection (depletion of CD8^+^ T cells abolished the protective effect of the vaccine). In addition to this clear role of CD8^+^ T cells, we found a moderate dependence on CD4^+^ T cells: depletion of this subset significantly reduced the protective efficacy of the vaccine, but compared to control naive mice, the CD4^+^ T cell-depleted mice showed low levels of protective efficacy, as measured by a 1-day median delay in the time to 1% parasitemia in the blood. Interestingly, the conserved epitope we previously identified which induced a CD4^+^ response (amino acids 51 to 80, covering part of the region predicted to be outside the membrane) is common to both BALB/c and C57BL/6 mice, in addition to HLA-A2-transgenic mice (20). It may be that this CD4^+^ epitope contributes to the consistent level of protection seen across different mouse strains.

There is now general consensus that an effective malaria vaccine will need to contain multiple antigens, either through whole-parasite vaccination or through inclusion of multiple antigens in a subunit vaccine (37). Viral vectors offer an effective platform for this purpose (38). However, evidence suggests that combining subunit vaccines can create competition or immune interference between antigens and reduce overall immunogenicity (39, 40). The competition seen is complex, and interference seems to be antigen dependent, as other studies have combined vaccines and reported no competition (41, 42). Research has also suggested that delivery of vaccines at separate sites can reduce antigen interference (39), but again, conflicting results have been reported (40). We found no significant decline or increase in cellular or humoral immunogenicity to either antigen when PfUIS3 and PfTRIP were administered alone or in combination (whether at separate sites or mixed) in BALB/c mice. Interestingly, coadministration of the two vaccines had a synergistic effect on protective efficacy. PfTRIP alone provided no protection, and PfUIS3 alone provided a delay in patent parasitemia, as expected, but coadministration (mixed) provided 100% sterile protection. This synergistic effect was evident only when the two vaccines were mixed and administered at the same site; the efficacy was only partially improved when the vaccines were administered at separate sites. As there was no significant change in immunogenicity detected in the blood for either vaccine upon coadministration, it is difficult to know the cause of the improved outcome. However, we did observe a trend toward higher PfTRAP-specific antibody responses (mixed vaccines or separate sites) and higher PfUIS3-specific CD8^+^ responses (mixed vaccines only), which may contribute to the synergistic effect observed. A study of coadministration of a virally vectored vaccine targeting internal antigens of the influenza virus with various protein formulations of the surface antigen hemagglutinin (HA) showed higher antibody responses against HA when the two vaccines were mixed rather than administered at separate sites (43) and attributed this to local effects in draining lymph nodes (an increase in the number of germinal center B cells). It will therefore be of interest to look at immunogenicity in our model at local sites, such as the draining lymph nodes, in addition to immunogenicity in the liver, given that it is the site mediating protection.

We previously identified PfUIS3-specific epitopes in HLA-A2-transgenic mice (including the conserved CD4^+^ epitope) (20), suggesting that human populations are also capable of inducing PfUIS3-specific immunity. We have now demonstrated that human populations do indeed respond to PfUIS3: cellular memory responses were identified in human volunteers who underwent CHMI with P. falciparum sporozoites. The median response of volunteers against PfUIS3 was greater than those observed against the four other preerythrocytic antigens tested (albeit not statistically significantly). In past studies of nonimmune humans exposed to sporozoites (whether for CHMI or for vaccination purposes), it has been difficult to pinpoint cellular responses to a particular antigen or antigens, including in our own recent work (23, 44). This is due to a combination of factors: T cell responses induced in humans following exposure to Plasmodium are low and are likely directed at a wide variety of antigens (44). One way to overcome the low responses recalled *ex vivo* is to use a cultured ELISpot assay to measure memory responses (45), as we have done in this study. Apart from their different sensitivities, the *ex vivo* and cultured ELISpot assays measure different cell populations: responses measured *ex vivo* reflect effector memory cells, while those measured after an extended culture period reflect central memory cells (24). Long-term memory is a goal of all vaccination strategies, and hence the ability to induce PfUIS3-specific central memory T cells in humans is promising for the use of this antigen in a multicomponent vaccine.

In conclusion, in this study we extended our recent findings to demonstrate that vaccination with ChAd63-MVA PfUIS3 can provide a consistent level of protection across multiple strains of mice and against challenge with chimeric P. berghei-P. falciparum parasites expressing PfUIS3, as well as WT P. berghei (expressing only PbUIS3). This protection is dependent upon the presence of CD8^+^ T cells, and to some extent CD4^+^ T cells, for which we previously identified a conserved epitope across multiple major histocompatibility complexes. We have also shown that humans who undergo CHMI with PfSPZ challenge develop central memory T cells against PfUIS3 and that administering PfUIS3 with PfTRIP in mice does not significantly reduce cellular immunogenicity to either antigen; in fact, combining both antigens results in 100% protection against double chimeric parasite challenge. While other antigens have been observed to provide greater levels of individual sterile protection preclinically, we have demonstrated the potential value of including PfUIS3 in a multicomponent malaria vaccine, particularly in combination with the PfTRAP antigen.

## MATERIALS AND METHODS

### Animals and ethics statement.

Female BALB/c (H-2^d^) or C57BL/6 (H-2^b^) mice of at least 6 weeks of age (Harlan, United Kingdom) were used in accordance with the UK Animals (Scientific Procedures) Act 1986 and approved by the University of Oxford Animal Care and Ethical Review Committee for use under protocol PPL 30/2414 or 30/2889. Animals were group housed in individually ventilated cages under specific-pathogen-free conditions. Temperature and humidity were kept constant, and a 12-h–12-h light-dark cycle was used. Short-term anesthesia was performed using vaporized IsoFlo, and all animals were humanely sacrificed at the end of experiments by an approved schedule 1 method (cervical dislocation). All efforts were made to minimize suffering. Carrying on from our previous studies, BALB/c mice were used as the primary strain of mice for immunogenicity and efficacy; C57BL/6 mice were used in one challenge experiment to demonstrate efficacy in another inbred strain of mice and in a separate experiment to measure CD8^+^ T cell responses in the liver.

### Vaccines, immunizations, and peptides.

The generation of the PfUIS3 vaccine has previously been described in detail (9). Briefly, the PfUIS3 vaccine is based on the 3D7 sequence, and no modifications were made apart from optimization to mammalian codon usage bias for expression in human cells (see Fig. S1 in the supplemental material). The regions of the UIS3 protein that are immunogenic in humans are currently unknown, and hence the whole protein is still under assessment. For the PfTRAP protein, we chose to use the vaccine denoted PfTRIP rather than the clinical ME-TRAP construct. This was for the following two reasons: first, the ME string contains the strong P. berghei Pb9 H-2^d^-restricted epitope from CSP (46), and hence this cannot be used to measure immunogenicity and efficacy directed at the TRAP protein in BALB/c mice; and second, the PfTRIP construct uses the 3D7 sequence (PlasmoDB accession no. PF3D7_1335900) (ME-TRAP uses the T9/96 strain sequence [47]), as does the PfUIS3 construct and the challenge parasites. The PfTRIP vaccine was codon optimized for expression in human cells and had 15 amino acids deleted, as does ME-TRAP (five repeats of PNP), as well as deletion of the predicted transmembrane helix and cytoplasmic domains (Fig. S1). The first 300 amino acids of the TRAP protein are known to be more immunogenic in humans than the transmembrane helix and cytoplasmic domains (48). Mice were immunized intramuscularly (i.m.) with 50 μl vaccine (in endotoxin-free Dulbecco's phosphate-buffered saline [D-PBS]) into the musculus tibialis, with doses stated in the relevant figure legends. When two vaccines were coadministered, full doses were administered individually into separate legs or mixed and administered into both legs (2 × 50 μl). All vaccines were administered using a ChAd63 prime followed 8 weeks later by an MVA boost. Overlapping peptide pools (synthetic crude 20-mers overlapping by 10 amino acids) were used to stimulate cells in the various immunoassays described below. The PfUIS3 pool contained a mix of 22 peptides (20), and the PfTRAP pool contained 50 peptides (Table S2). Each individual peptide was originally dissolved to a concentration of either 50 or 100 mg/ml prior to pooling of the peptides for use in *in vitro* restimulation at a final peptide concentration of 5 μg/ml. The dimethyl sulfoxide (DMSO) concentration was always less than 1% in the final peptide pool. Peptides were purchased from Neo Group Inc. or Thermo Fisher Scientific.

### ICS assay.

An intracellular cytokine staining (ICS) assay was used to detect antigen-specific cellular responses in the blood, spleen, and liver and was performed as previously described (9). Briefly, after lysis of red blood cells, single-cell suspensions were stimulated for 6 h with a final concentration of 5 μg/ml of the appropriate peptide pool (PfUIS3 or PfTRAP), 1 μg/ml GolgiPlug (BD Biosciences, United Kingdom), and anti-mouse CD107a–phycoerythrin (PE). Unstimulated wells without peptide were used as background controls. For standard ICS, cells were surface stained with anti-mouse CD16/32, CD4-eFluor 450, and CD8α–peridinin chlorophyll protein (PerCP)–Cy5.5 antibodies, and intracellular cytokines were stained with tumor necrosis factor alpha (TNF-α)–fluorescein isothiocyanate (FITC), IL-2–PE–Cy7, and IFN-γ–allophycocyanin (APC) antibodies diluted in Perm/Wash reagent (BD Biosciences). The gating strategy and representative plots are shown in Fig. S2 in the supplemental material. To stain for memory cell markers, the first layer contained anti-mouse CD16/32, CD8α-PerCP-Cy5.5, CD4-eFluor 650, CD62L-PE-Cy7, and CD127-APC-eFluor 780 antibodies and Live/Dead Aqua reagent. The second layer contained anti-mouse TNF-α–FITC and IFN-γ–eFluor 450 antibodies diluted in Perm/Wash reagent. The gating strategy used for memory cell markers was published previously (9). Data were acquired using an LSRII flow cytometer (BD Biosciences) and analyzed using FlowJo (Tree Star Inc.). All antibodies were purchased from BD Biosciences, eBioscience, or Invitrogen, United Kingdom.

### Antibody measurements.

IgG antibody responses were measured using the luminescence immunoprecipitation system (LIPS) as previously described (9).

### Parasites and efficacy studies.

Sporozoites were obtained by dissection and homogenization of salivary glands from Anopheles stephensi mosquitoes at 21 days postinfection. Blood stocks of green fluorescent protein (GFP)-expressing P. berghei parasites (49) were kindly provided by Robert Sinden (Imperial College, London, and Oxford University). The generation of chimeric P. berghei-P. falciparum parasites expressing PfUIS3 has been described previously (9). In addition, we generated double chimeric P. berghei-P. falciparum parasites that express both PfUIS3 and PfTRAP under the control of the *Pbuis4* promoter (see below). One thousand sporozoites were injected intravenously (i.v.) into the tail vein 8 to 10 days following the final vaccination, as detailed in the relevant figure legends. Mice were monitored from 4 days postinfection by use of Giemsa-stained thin-film blood smears. The experimental endpoint was 14 or 15 days parasite free (sterilely protected) or when blood-stage parasites had been confirmed on three consecutive days. The time to 1% parasitemia was calculated using linear regression; when sterile protection is not achieved, this value is adept at providing a sensitive measure of liver-stage protection, as it reflects the number of parasites erupting from the liver (50, 51). In some experiments, subsets of T cells were depleted using the monoclonal antibody (MAb) anti-CD4 clone GK1.5 or anti-CD8 clone 2.43 (both are rat IgG2a antibodies purified by protein G affinity chromatography from hybridoma culture supernatants). An IgG control (Sigma-Aldrich) was purified using the same method. One hundred micrograms of antibody injected 2 days before and on the day of challenge successfully depleted 100% of either cell population (9), and this depletion was maintained until at least 4 days postchallenge. The degree of *in vivo* CD4^+^ or CD8^+^ T cell depletion was assessed by flow cytometry using 1/100 anti-CD4–FITC clone RM4-5, 1/200 anti-CD8–PerCP–Cy5.5 clone 53-6.7, and 1/50 anti-CD3ε–APC.

### Generation of P. berghei-P. falciparum double chimeric parasites expressing both PfUIS3 and PfTRAP.

To generate the double additional gene (DAG) chimeric parasite PbANKA-PfTRAP+PfUIS3@Pbuis4 DAG (line 2395cl1), we used the previously generated single additional gene (SAG) PbANKA-PfTRAP_Pbuis4_ line (line 2281cl1) as the background parent line (9). This line expresses the PfTRAP (accession no. PF3D7_1335900) coding sequence (CDS) under the control of the *Pbuis4* regulatory sequences and a fusion protein of GFP and firefly luciferase (LUC-IAV) under the control of the constitutive *Pbeef1a* promoter. This transgenic parasite is selectable marker (SM) free. Both the *trap* and *Luc-gfp* expression cassettes are integrated into the neutral *230p* locus in chromosome 3. In this line, the PfUIS3 CDS (PfUIS3; accession no. PF3D7_1302200) was stably integrated into a neutral *s1* gene locus (*Pbs1*; accession no. PBANKA_120680) (52, 53; A. M. Salman and C. J. Janse, unpublished data) through double-crossover recombination using a 2-step “gene insertion/marker out” (GIMO) transfection protocol (52, 54) (Fig. S3). In the first step, we deleted the Pbs1 CDS and replaced it with a positive-negative selectable marker to create a *Pbs1* deletion GIMO line (PbANKA-PfTRAP+PbΔs1 GIMO; line 2353cl2). In order to do this, we generated the pL1928 construct, based on the standard GIMO DNA construct pL0034 (54). This construct contains a positive-negative (h*dhfr*::y*fcu*) SM cassette and was used to insert both the *Pbs1* 5′ and 3′ gene targeting regions (TRs) (52). The linear pL1928 DNA construct was introduced into PbANKA-PfTRAP_Pbuis4_ parasites (line 2281cl1) (9) by using standard methods of transfection (55). Transfected parasites were selected in mice through addition of pyrimethamine in the drinking water (55). Transfected parasites were cloned by limiting dilution (56), resulting in the PbANKA-PfTRAP+PbΔs1 GIMO line (line 2353cl2). Correct deletion of the Pbs1 CDS and its replacement with the SM cassette were confirmed by diagnostic PCR analysis of genomic DNA (gDNA) and Southern analysis of pulsed-field gel electrophoresis-separated chromosomes as described previously (9) (Fig. S4). Primers used for PCR genotyping are listed in Table S2.

In the second step, we replaced the positive-negative SM in the genome of the PbANKA-PfTRAP+PbΔs1 line (line 2353cl2) with the PfUIS3 CDS by GIMO transfection to create a P. berghei DAG chimeric line expressing PfTRAP and PfUIS3. This was achieved by modifying the construct used in the first step (pL1928); specifically, the h*dfhr*::y*fcu* SM cassette was removed and replaced with the PfUIS3 CDS expression cassette, generating plasmid pL2043. The expression cassette in pL2043 contained the PfUIS3 CDS flanked by the 5′ and 3′ regulatory sequences of *Pbuis4*, which were amplified from P. berghei ANKA WT genomic DNA. The PfUIS3 CDS was amplified from genomic DNA of the NF54 strain of P. falciparum. The pL2043 construct was sequenced to ensure that there were no mutations in the PfUIS3 CDS. The construct was linearized using the HindIII restriction enzyme sites outside the 5′ and 3′ Pbs1 TRs before transfection. The construct was used to transfect parasites of the PbANKA-PfTRAP+PbΔs1 GIMO line (line 2353cl2) by using standard methods of GIMO transfection (54). Transfected parasites were selected in mice by applying negative selection by providing 5-fluorocytosine (5FC) in the drinking water (57). This resulted in selection of chimeric parasites in which the h*dhfr*::y*fcu* SM in the *Pbs1* locus of the PbANKA-PfTRAP+PbΔs1 GIMO line was replaced by the PfUIS3 CDS expression cassette. Selected chimeric parasites were cloned by limiting dilution (56). Correct integration of the constructs into the genome of chimeric parasites was analyzed by diagnostic PCR analysis of gDNA and Southern analysis of pulsed-field gel electrophoresis-separated chromosomes. Primers used for PCR genotyping are listed in Table S1. This method created DAG chimeric P. berghei-P. falciparum parasites that express full-length PfTRAP and PfUIS3 under the control of the *Pbuis4* regulatory sequences and are SM free.

### Phenotyping of chimeric parasites.

Multiplication of blood stages in mice was determined during the cloning period as described previously (58). Feeding of A. stephensi mosquitoes for determination of oocyst and sporozoite numbers was performed as described previously (9). Infection dynamics of the double chimeric parasites in mice following sporozoite injection were equivalent to those of WT P. berghei parasites (Fig. S5).

### CHMI with PfSPZ challenge and cultured IFN-γ ELISpot assay.

Cryopreserved human peripheral blood mononuclear cells (PBMCs) were obtained from a previously published clinical trial of the Sanaria PfSPZ challenge in malaria-naive UK adults (trial no. NCT01465048 at ClinicalTrials.gov) (23). In brief, 18 healthy volunteers received aseptic, purified, cryopreserved, live, infectious P. falciparum sporozoites (PfSPZ challenge [59]) (2,500 or 25,000) injected either i.m. or intradermally. A total of 14/18 volunteers had parasites identified in the blood by quantitative PCR performed postinjection. Cryopreserved PBMCs from 35 days postinjection were used to assess T cell memory responses in a cultured IFN-γ ELISpot assay (24). A total of 5 × 10^6^ thawed cells were added to the middle wells of a 12-well plate, along with 4 μg/ml of the appropriate peptide pool, and incubated at 37°C with 5% CO_2_. On days 3 and 7, half the medium volume was replaced with recombinant human IL-2 (final concentration, 25 IU/ml). On day 9, cells were washed to remove all traces of IL-2 and left to rest overnight. On day 10, the IFN-γ ELISpot assay was performed as previously described (23). Briefly, cells were incubated for 18 to 20 h with 20 μg/ml of the appropriate overlapping peptide pool in ELISpot plates (Mabtech, Sweden) coated with anti-IFN-γ. After addition of the detecting antibody and substrate, spots were enumerated using an ELISpot plate counter (AID, Germany). All wells were set up in quadruplicate. Due to the higher background and variability in a cultured versus *ex vivo* ELISpot assay, a stringent positivity cutoff was set by performing the assay using cryopreserved PBMCs taken from volunteers the day prior to controlled human malaria infection (CHMI) with PfSPZ challenge. This was performed for four volunteers with two different antigenic stimulations, and the positivity cutoff was set as the overall mean plus 2 standard deviations.

### Statistical analysis.

The statistical software Prism, version 6 (GraphPad), was used for all analyses. Survival in challenge experiments is presented using Kaplan-Meier curves, and significance was tested using the log-rank (Mantel-Cox) test. The significance threshold was 0.05. Nonparametric data are shown as medians with individual data points plotted unless otherwise indicated.

## Supplementary Material

Supplemental material
