# Supplementary material for: Assessment of the Plasmodium falciparum Preerythrocytic Antigen UIS3 as a Potential Candidate for a Malaria Vaccine
Source: Infect Immun. 2017 Feb 23;85(3):e00641-16. doi: 10.1128/IAI.00641-16 (PMC5328496; doi:10.1128/IAI.00641-16)
Supplement: Supplemental material [file supp_85_3_e00641-16__index.html]

Supplemental material 

# Assessment of the Plasmodium falciparum Preerythrocytic Antigen UIS3 as a Potential Candidate for a Malaria Vaccine

## Supplemental material

- Supplemental file 1 -

  Fig. S1. Schematic diagram of the antigens PfUIS3 and PfTRAP, with reference to the vaccine constructs. Fig. S2. Gating strategy used for ICS experiments. Fig. S3. Generation of the DAGs chimeric parasite line PfTRAP+PfUIS3@Pbuis4 (line 2395cl1). Fig. S4. Genotype analysis of the DAGs chimeric parasite line PfTRAP+PfUIS3@Pbuis4 (line 2395cl1) and its intermediate GIMO mother-line (2353cl2). Fig. S5. Infectivity of the double chimeric parasites in BALB/c mice compared to WT Pb. Fig. S6. PfCelTOS and PfFalstatin do not protect against WT Pb challenge. Table S1. PfTRAP (3D7) peptide sequences. Table S2. Primers for genotyping the DAGs chimeric parasite line 2395cl1.

  PDF, 1.1M
