# Supplementary material for: Assessment of the Plasmodium falciparum Preerythrocytic Antigen UIS3 as a Potential Candidate for a Malaria Vaccine
Source: Infect Immun. 2017 Feb 23;85(3):e00641-16. doi: 10.1128/IAI.00641-16 (PMC5328496; doi:10.1128/IAI.00641-16)
Supplement: Supplemental material [file IAI.00641-16_zii999091979s1.pdf]

SUPPLEMENTAL MATERIAL

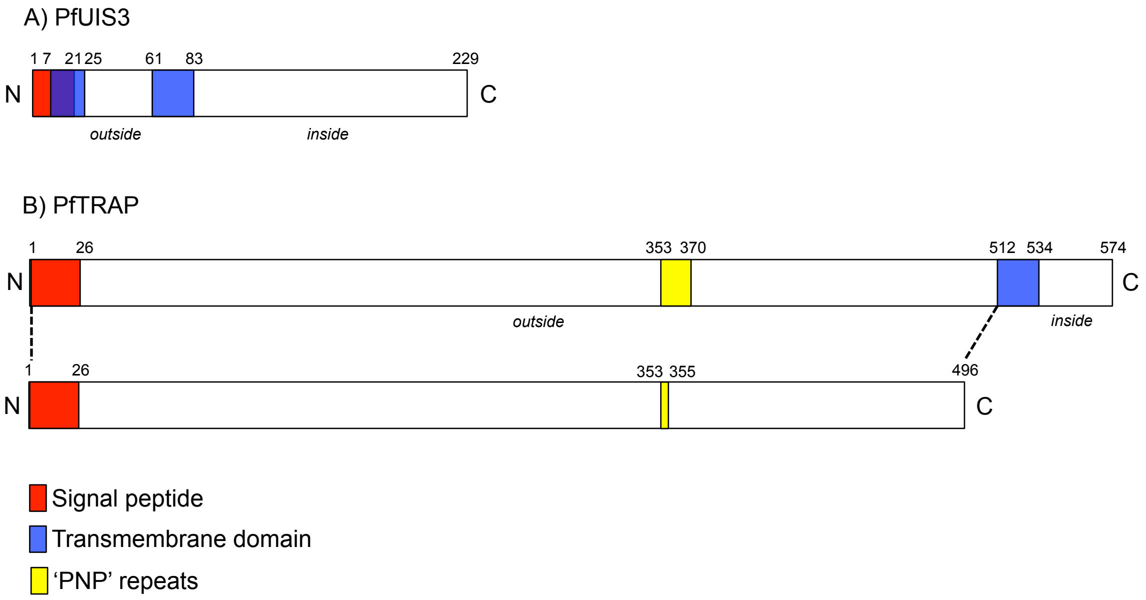

FIG S1 Schematic diagram of the antigens PfUIS3 and PfTRAP, with reference to the vaccine constructs. The protein sequence of PfUIS3 used for the vaccine construct was identical to that of Pf 3D7 UIS3 (PF3D7_1302200). Protein features were obtained from PlasmoDb and the TMHMM server (v.2.0) from the Center for Biological Sequence Analysis (http://www.cbs.dtu.dk/services/). The protein sequence of PfTRAP (PF3D7_1335900) used for the vaccine construct PfTRIP had a number of modifications. The first schematic shows the full PfTRAP 3D7 sequence from PlasmoDB, whilst the second schematic shows the modified vaccine construct. The protein features of TRAP were obtained from a previous publication (1). The vaccine construct does not include the predicted transmembrane and cytoplasmic domains (amino acids 512-574), and contains only one of the 'PNP' repeats.

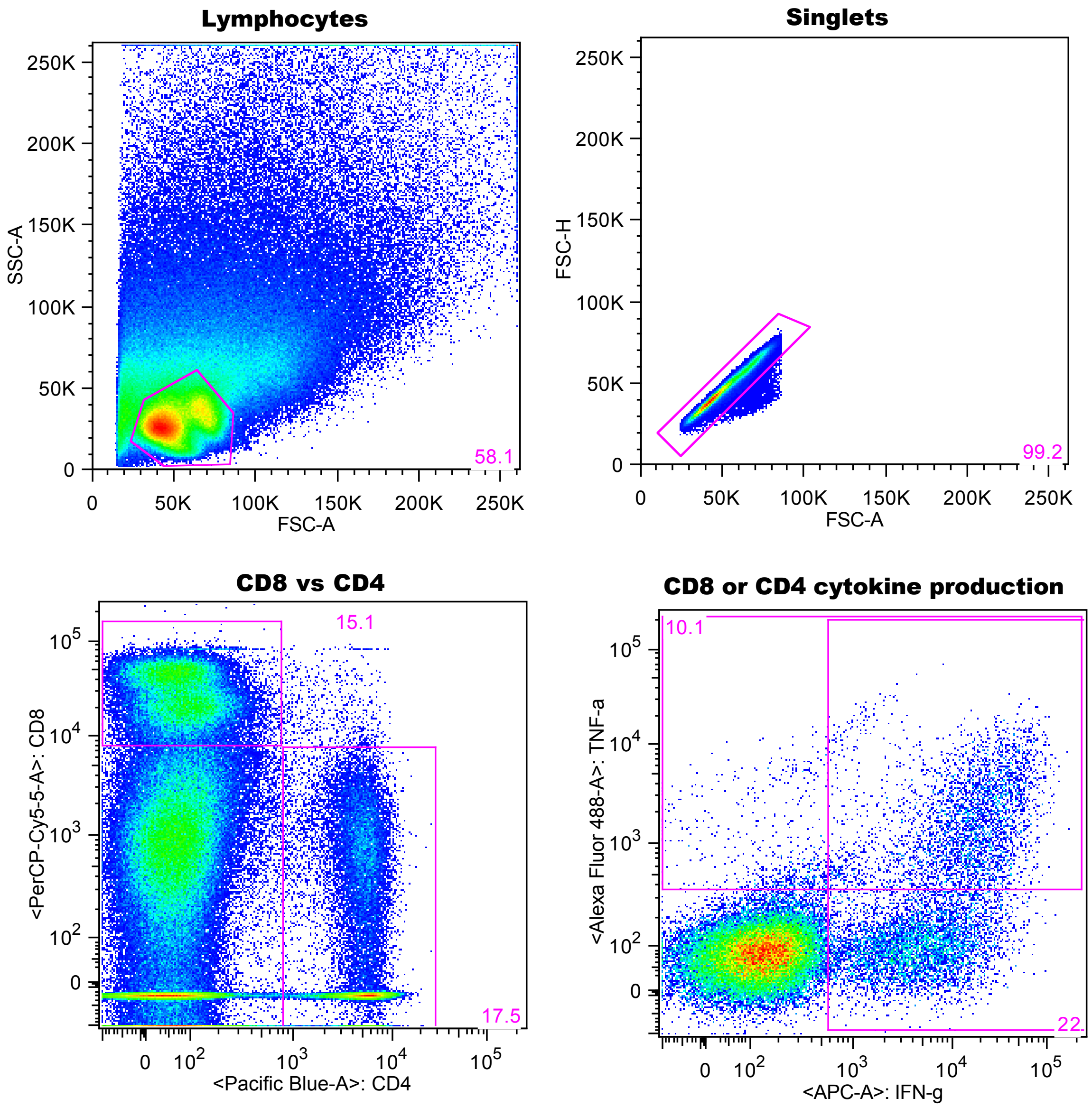

FIG S2 Gating strategy used for ICS experiments. Cell suspensions from the spleen or blood were first gated by size, followed by singlet cells. The cells were then separated into CD4 or CD8 positive subsets, and then cytokines gated from within those subsets. Gates show the percentage of the parent.

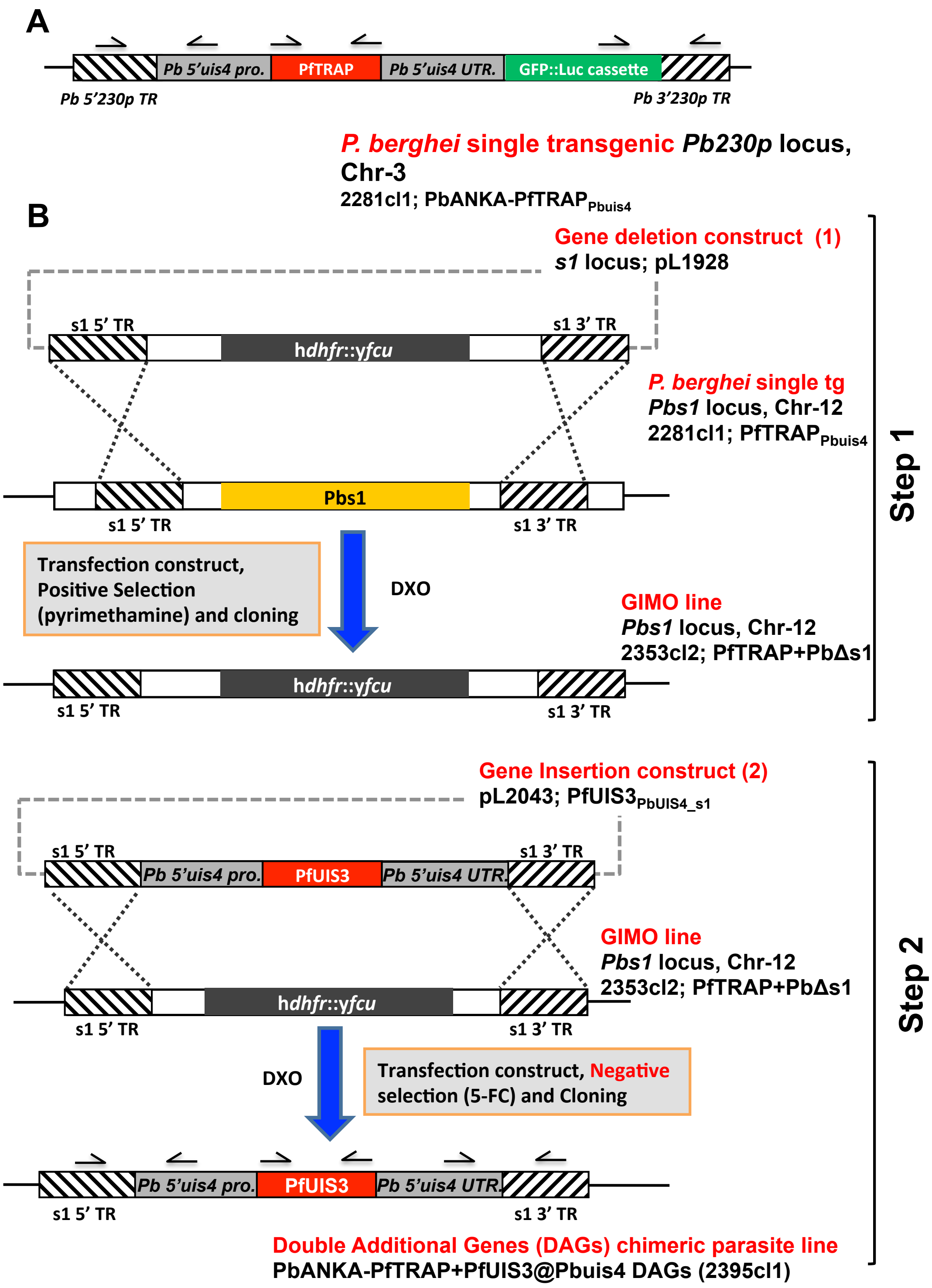
A
Pb 5'uis4 pro.
PfTRAP
Pb 5'uis4 UTR.
GFP::Luc cassette
Pb 5'230p TR
Pb 3'230p TR
P. berghei single transgenic Pb230p locus, Chr-3
2281cl1; PbANKA-PfTRAP$_{Pbuis4}$
B
Gene deletion construct (1)
s1 locus; pL1928
s1 5' TR
hdhfr::yfcu
s1 3' TR
P. berghei single tg
Pbs1 locus, Chr-12
2281cl1; PfTRAP$_{Pbuis4}$
Pbs1
s1 5' TR
s1 3' TR
Transfection construct, Positive Selection (pyrimethamine) and cloning
DXO
GIMO line
Pbs1 locus, Chr-12
2353cl2; PfTRAP+PbΔs1
hdhfr::yfcu
s1 5' TR
s1 3' TR
Step 1
Gene Insertion construct (2)
pL2043; PfUIS3$_{PbUIS4_s1}$
s1 5' TR
Pb 5'uis4 pro.
PfUIS3
Pb 5'uis4 UTR.
s1 3' TR
GIMO line
Pbs1 locus, Chr-12
2353cl2; PfTRAP+PbΔs1
hdhfr::yfcu
s1 5' TR
s1 3' TR
DXO
Transfection construct, Negative selection (5-FC) and Cloning
Pb 5'uis4 pro.
PfUIS3
Pb 5'uis4 UTR.
s1 5' TR
s1 3' TR
Double Additional Genes (DAGs) chimeric parasite line
PbANKA-PfTRAP+PfUIS3@Pbuis4 DAGs (2395cl1)
Step 2

FIG S3 Generation of the DAGs chimeric parasite line PfTRAP+PfUIS3@Pbuis4 (line 2395cl1). (A) Schematic representation of the previously generated PfTRAP$_{\text{Pbuis4}}$ (2281cl1) (2)**.** This SAG transgenic parasite line was used as the background parent line for the generation of DAG chimeric parasite PfTRAP+PfUIS3@Pbuis4 (line 2395cl1; see B). It expresses the PfTRAP CDS under the control of the *Pbuis4* regulatory sequences and a fusion protein of GFP and LUC-IAV under the constitutive *Pbeef1a* promoter. This transgenic parasite is SM free. Both the *trap* and *Luc-gfp* expression cassettes were integrated into the neutral *230p* locus in chromosome 3. (B) Schematic representation of the generation of the DAGs chimeric parasite PbANKA-PfTRAP+PfUIS3@Pbuis4 (line 2395cl1), where the *Pbs1* neutral locus (PBANKA_120680) in chromosome 12 in the PfTRAP$_{\text{Pbuis4}}$ (2281cl1; see A) SAG transgenic parasite line was replaced with the second Pf gene expression cassette PfUIS3$_{\text{Pbuis4_s1}}$. 1st step: The GIMO deletion construct (Construct 1; pL1928) was used to replace the *Pbs1* CDS with the positive/negative selectable maker (h*dhfr::*y*fcu*) cassette, resulting in the generation of the Pbs1 GIMO line (*Pb*ANKA-PfTRAP+*Δs1*; 2353cl2) after positive selection with pyrimethamine. Construct 1 targets the *Pbs1* gene by double cross-over homologous recombination. After genotyping and confirmation of correct construct integration, this line was cloned by limiting dilution. 2nd step: The GIMO insertion construct (construct 2) was used to replace the SM in the *PbΔs1* GIMO line (*Pb*ANKA-PfTRAP+*Δs1*; 2353cl2) with the second Pf gene expression cassette PfUIS3$_{\text{PbUIS4_s1}}$, under negative (5-FC) selection. Construct 2 integrates by double cross-over homologous recombination using the same TRs employed in construct 1, resulting in the introduction of the Pf gene under the control of *Pbuis4* regulatory sequences. Transfection, negative selection and cloning

results in the DAGs chimeric parasite PfTRAP+PfUIS3@Pbuis4 (line 2395cl1). This line contains both the Pf *trap* and *uis3* genes under the control of the *Pbuis4* regulatory sequences and is SM free. Black arrows: location of primers used for diagnostic PCR (see Table S1).

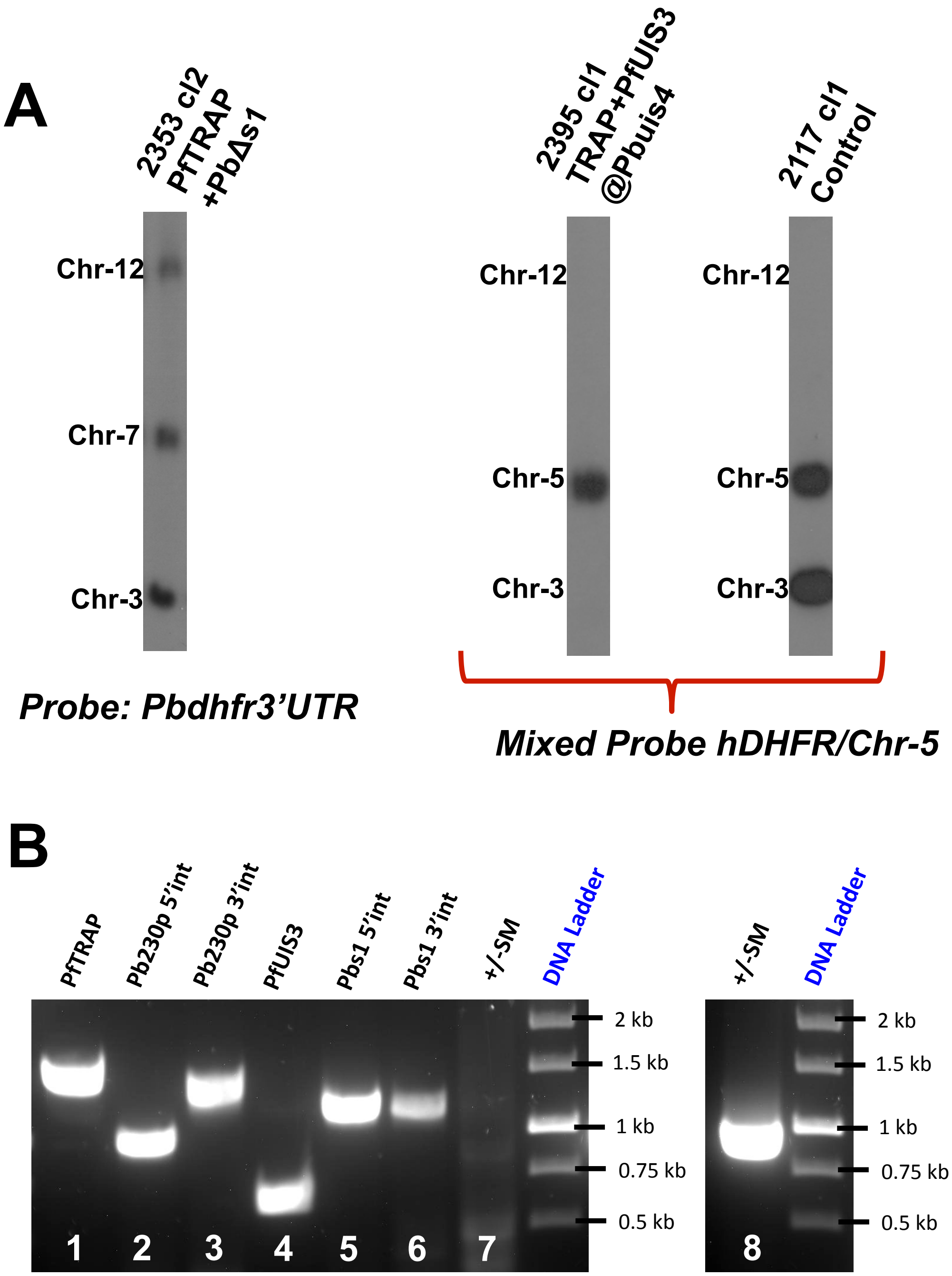

Lane	*PCR product*	*Primers*		*Size (bp)*
		F	*R*	
1	PfTRAP	*1106*	*1107*	*1,727*
2	Pb230p 5'int	*1080*	*1081*	*1,100*
3	Pb230p 3'int	*1051*	*1083*	*1,602*
4	PfUIS3	*1096*	*1097*	*691*
5	Pbs1 5'int	*1127*	*1081*	*1,309*
6	Pbs1 3'int	*1082*	*1128*	*1,308*
7	+/-SM	*1048*	*1049*	*NA*
8	*+/-SM Control*	*1048*	*1049*	*927*

FIG S4 Genotype analysis of the DAGs chimeric parasite line PfTRAP+PfUIS3@Pbuis4 (line 2395cl1) and its intermediate GIMO mother-line (2353cl2). (A) Hybridisation of chromosomes (chr.) of line 2353cl2 (left panel) with the 3′UTR *Pbdhfr/ts* confirms integration of construct pL1928 (Fig. S2) into the *Pbs1* gene on chr. 12. In addition, this probe hybridizes to the GFP-Luc reporter-cassette in chr. 3 (Fig. S2) and to the endogenous *Pbdhfr/ts* on chr. 7. The correct integration of the PfUIS3 expression construct (pL2043; Fig. S2) into the Pbs1 GIMO locus was confirmed by showing the removal of the h*dhfr::*y*fcu* SM cassette in the cloned chimeric parasite line 2395cl1 (right panel) . The southern blot was hybridized with a mixture of two probes: one recognizing h*dhfr* and a control probe recognizing chr. 5. As an additional control, parasite line 2117cl1 was used with the h*dhfr::*y*fcu* SM integrated into chr. 3. (B) Genotype analysis by diagnostic PCR of the chimeric parasite line 2395cl1 (left panel) confirmed correct integration of both PfTRAP and PfUIS3 expression cassettes. Correct integration is shown by the absence of the h*dhfr*::y*fcu* SM, the presence of the PfTRAP CDS and PfUSI3 CDS, and the correct integration of the PfTRAP and PfUIS3 constructs into the genome both at the 5’ and 3’regions of Pb230 and Pbs1 loci, respectively (5’int and 3’int; see Fig. S2 for primer locations). Primers sequences used are shown in Table S1.

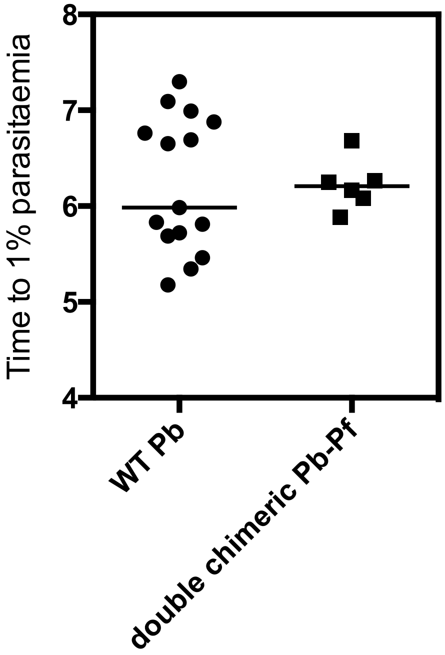

FIG S5 Infectivity of the double chimeric parasites in BALB/c mice compared to WT Pb. Time to 1% parasitaemia of the double chimeric Pb-Pf parasites (n=6) compared to WT Pb (n=15) following injection of 1000 sporozoites i.v. There was no statistical difference between the two groups, Mann-Whitney test p=0.85. Data for the WT Pb is the same as used in Figure S6 of our previous manuscript (2), to enable direct comparison with the single chimeric Pb-Pf parasites for UIS3 and TRAP.

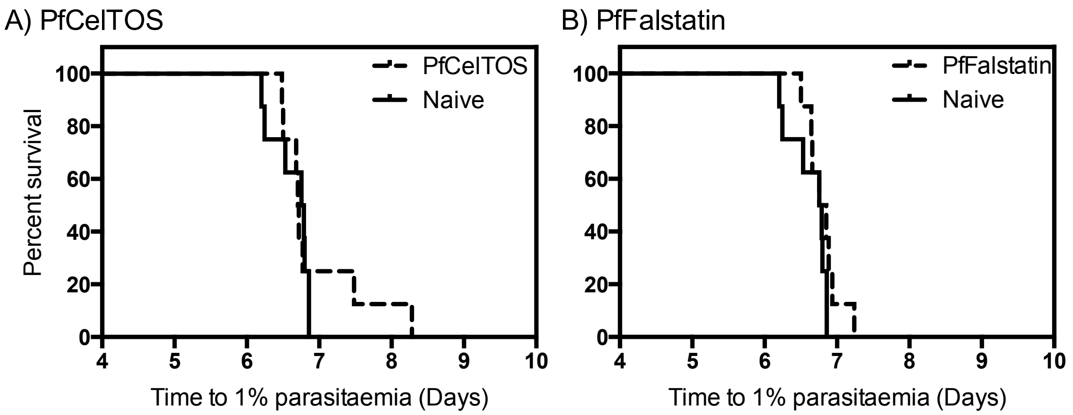

FIG S6 PfCelTOS and PfFalstatin do not protect against WT Pb challenge. BALB/c mice (n=8) were vaccinated i.m. with $1x10^8$ ifu ChAd63 followed eight weeks later by $1x10^7$ pfu MVA, either against (A) PfCelTOS or (B) PfFalstatin. Eight days post-boost mice were challenged i.v. with 1000 Pb sporozoites, along with eight naïve control mice. The Log-rank (Mantel-Cox) Test was used to assess the difference between the survival curves: (A) p=0.32, (B) p=0.19.

TABLE S1 PfTRAP (3D7) peptide sequences. The PfUIS3 peptides used have been previously published, see Table S2 in (3).

Peptide	Sequence
TRAP-1	MNHLGNVKYLVIVFLIFFDL
TRAP-2	VIVFLIFFDLFLVNGRDVQN
TRAP-3	FLVNGRDVQNNIVDEIKYRE
TRAP-4	NIVDEIKYREEVCNDEVDLY
TRAP-5	EVCNDEVDLYLLMDCSGSIR
TRAP-6	LLMDCSGSIRRHNWVNHAVP
TRAP-7	RHNWVNHAVPLAMKLIQQLN
TRAP-8	LAMKLIQQLNLNDNAIHLYA
TRAP-9	LNDNAIHLYASVFSNNAREI
TRAP-10	SVFSNNAREIIRLHSDASKN
TRAP-11	IRLHSDASKNKEKALIIIKS
TRAP-12	KEKALIIIKSLLSTNLPYGK
TRAP-13	LLSTNLPYGKTNLTDALLQV
TRAP-14	TNLTDALLQVRKHLNDRINR
TRAP-15	RKHLNDRINRENANQLVVIL
TRAP-16	ENANQLVVILTDGIPDSIQD
TRAP-17	TDGIPDSIQDSLKESRKLSD
TRAP-18	SLKESRKLSDRGVKIAVFGI
TRAP-19	RGVKIAVFGIGQGINVAFNR
TRAP-20	GQGINVAFNRFLVGCHPSDG

TRAP-21	FLVGCHPSDGKCNLYADSAW
TRAP-22	KCNLYADSAWENVKNVIGPF
TRAP-23	ENVKNVIGPFMKAVCVEVEK
TRAP-24	MKAVCVEVEKTASCGVWDEW
TRAP-25	TASCGVWDEWSPCSVTCGKG
TRAP-26	SPCSVTCGKGTRSRKREILH
TRAP-27	TRSRKREILHEGCTSELQEQ
TRAP-28	EGCTSELQEQCEEERCLPKR
TRAP-29	CEEERCLPKREPLDVPDEPE
TRAP-30	EPLDVPDEPEDDQPRPRGDN
TRAP-31	DDQPRPRGDNFAVEKPNENI
TRAP-32	FAVEKPNENIIDNNPQEPSP
TRAP-33	IDNNPQEPSPNPEEGKGENP
TRAP-34	NPEEGKGENPNGFDLDENPE
TRAP-35	NGFDLDENPENPPNPPNPPN
TRAP-36	NPPNPPNPPNPPNPPNPPNP
TRAP-37	PPNPPNPPNPDIPEQKPNIP
TRAP-38	DIPEQKPNIPEDSEKEVPSD
TRAP-39	EDSEKEVPSDVPKNPEDDRE
TRAP-40	VPKNPEDDREENFDIPKKPE
TRAP-41	ENFDIPKKPENKHDNQNNLP
TRAP-42	NKHDNQNNLPNDKSDRYIPY
TRAP-43	NDKSDRYIPYSPLAPKVLDN

TRAP-44	SPLAPKVLDNERKQSDPQSQ
TRAP-45	ERKQSDPQSQDNNGNRHVPN
TRAP-46	DNNGNRHVPNSEDRETRPHG
TRAP-47	SEDRETRPHGRNNENRSYNR
TRAP-48	RNNENRSYNRKHNNTPKHPE
TRAP-49	KHNNTPKHPEREEHEKPDNN
TRAP-50	REEHEKPDNNKKKAGSDNKY

TABLE S2 Primers for genotyping the DAGs chimeric parasite line 2395cl1

Primer	Description	Primer sequences
1048	hDHFR-yFCU (+/-SM) F	ATCATGCAAGACTTTGAAAGTGAC
1049	hDHFR-yFCU (+/-SM) R	CATCGATTCACCAGCTCTGAC
1051	Luciferase F	GTCGCCAGTCAAGTAACAAC
1080	Pb5'230p Integration F	ACTGTTATATTTGGTGATGGAATGG
1081	Pb5'230p Integration R	TATACATCCACGGATGCATAGAAG
1082	Pb3'230p Integration F	TCTGCATTAACCTTAAATATGAAAAACAC
1083	Pb3'230p Integration R	TTCAGTGAAATCGCAAACATAAGTATC
1096	PfUIS3 F	AATGAAGGTCTCTAAATTAGTCTTG
1097	PfUIS3 R	TTAGTTCTCTTCTTGAGATAAATAATTAGCATAAAC
1106	PfTRAP F	ATGAATCATCTTGGGAATGTTAAATATTTAGTC
1107	PfTRAP R	ATTTAATTCCACTCGTTTTCTTCAGG
1127	Pb3'S1 Integration R	TGTACTACTTTCACATCAAATTCAGTAACC
1128	Pb5'S1 Integration F	TAAAGGTTAGCATTCAATCTGTCTG

REFERENCES

1. **Pihlajamaa T, Kajander T, Knuuti J, Horkka K, Sharma A, Permi P.** 2013. Structure of Plasmodium falciparum TRAP (thrombospondin-related anonymous protein) A domain highlights distinct features in apicomplexan von Willebrand factor A homologues. Biochem J **450:**469-476.
2. **Longley RJ, Salman AM, Cottingham MG, Ewer K, Janse CJ, Khan SM, Spencer AJ, Hill AV.** 2015. Comparative assessment of vaccine vectors

encoding ten malaria antigens identifies two protective liver-stage candidates. Sci Rep **5:**11820.

3. **Longley RJ, Halbroth BR, Ewer KJ, Hill AV, Spencer AJ.** 2015. Identification of Immunodominant Responses to the Plasmodium falciparum Antigens PfUIS3, PfLSA1 and PfLSAP2 in Multiple Strains of Mice. PLoS One **10:**e0144515.
